# Differences in the Properties of the Radial Artery between *Cun*, *Guan*, *Chi*, and Nearby Segments Using Ultrasonographic Imaging: A Pilot Study on Arterial Depth, Diameter, and Blood Flow

**DOI:** 10.1155/2015/381634

**Published:** 2015-02-11

**Authors:** Jaeuk U. Kim, Yu Jung Lee, Jeon Lee, Jong Yeol Kim

**Affiliations:** ^1^Medical Engineering R&D Group, Korea Institute of Oriental Medicine, Daejeon 305-811, Republic of Korea; ^2^Johns Hopkins University School of Medicine, Baltimore, MD 21205-2196, USA

## Abstract

*Aim of the Study*. The three conventional pulse-diagnostic palpation locations (PLs) on both wrists are *Cun*, *Guan*, and *Chi*, and each location reveals different clinical information. To identify anatomical or hemodynamic specificity, we used ultrasonographic imaging to determine the arterial diameter, radial artery depth, and arterial blood flow velocity at the three PLs and at nearby non-PL segments. *Methods*. We applied an ultrasound scanner to 44 subjects and studied the changes in the arterial diameter and depth as well as in the average/maximum blood flow velocities along the radial artery at three PLs and three non-PLs located more proximally than *Chi*. *Results*. All of the measurements at all of the PLs were significantly different (*P* < 0.01). Artery depth was significantly different among the non-PLs; however, this difference became insignificant after normalization to the arm circumference. *Conclusions*. Substantial changes in the hemodynamic and anatomical properties of the radial artery around the three PLs were insignificant at the nearby non-PLs segments. This finding may provide a partial explanation for the diagnostic use of “*Cun*, *Guan*, and *Chi.*”

## 1. Introduction

Pulse diagnosis is one of the most important diagnostic methods in traditional East Asian medicine (TEAM) [1–3]. By diagnosing the pulse, trained practitioners can reveal information on the balance of* Qi* and blood and on the homeostasis of the body and various organ systems [1]. Historically, the pulse was assessed at various regions of the body, including the carotid artery, temporal artery, dorsalis pedis, femoral and popliteal arteries, and radial arteries [1]. In contemporary TEAM, the pulse is commonly diagnosed using the radial arteries of both wrists. These regions of the wrist comprise three distinct palpation locations (PLs) that span 5 cm along the radial artery:* Cun (Chon), Guan (Gwan)*, and* Chi (Cheok)*.

A shallow-lying artery that is firmly supported by the prominent bone and ligament characterizes the three PLs regions; however, the bulge of the prominent bone should not be confused with the nearby styloid process [4]. To determine the palpation locations, a practitioner places his/her middle finger on the prominent bone and sequentially lines up the index and ring fingers on the radial artery [1, 4].* Guan* is located just below the middle finger,* Cun* below the index finger, and* Chi* beneath the ring finger.

The exact locations of* Cun* and* Chi* vary with the patient's elbow length (EL). In accordance with ancient TEAM scriptures, Tyan et al. showed that the lengths of* Cun, Guan*, and* Chi* in a cohort of 200 adult subjects were approximately 6%, 6%, and 7% of the EL, respectively [5]; the 6% of the EL was approximately 1.54 cm for male subjects and 1.4 cm for female subjects. This result reflects the varying elbow lengths between the two gender groups. Similarly, a recent survey of 78 adult subjects showed that* Cun* was approximately 1.14 cm distal from* Guan* and* Chi* was approximately 1.49 cm proximal from* Guan* [6]. Both studies showed that* Cun, Guan,* and* Chi* were located adjacent to one another.

The pulses at different PLs are believed to contain specific clinical information on body parts and organ systems [3, 7, 8]. For instance, the pulse at* Cun* describes the organ function within the upper region of the trunk and thoracic cavity, such as that of the lungs and heart; the pulse at* Guan* describes the condition of the upper abdominal cavity, which includes the liver, spleen, and pancreas; and the pulse at* Chi* reflects the condition of the lower abdominal cavity, which includes the urinary and reproductive organs. The balance of the pulses felt between* Cun, Guan*, and* Chi* reflects the balance between the corresponding organ functions in the upper, middle, and lower body parts. Similarly, the pulses between the left and right wrists describe the balance between the left and right parts of the body.

How is it possible that pulses at three PLs located on the same arterial segment with the same blood flowing through them contain such distinct clinical information? The correspondence hypothesis between the palpation locations and the organ systems has been advocated by a long history of clinical experience. However, it is a scientific challenge to verify and understand the physiological principles of this hypothesis.

Here, as an initial stage for exploring the above fundamental question, we study some anatomical and hemodynamic properties of the radial artery at three PLs and nearby segments; studying nearby segments would help us understand more clearly the distinct features of the PLs. For this purpose, we use ultrasonography of healthy subjects to investigate the geometric properties of the radial artery and blood flow. We compare these properties at the three PLs and at three non-pulse-diagnostic locations (non-PLs) that are more proximal than* Chi* on the same radial artery and the left versus right arms.

## 2. Methods

The study was carried out with the approval of the Institutional Review Board (IRB, djomc-18) of the Oriental Hospital of Daejeon University. The basic health conditions of each subject were first examined by traditional Korean medical doctors (TKMDs) using blood pressure measurements and cholesterol tests. Forty-four healthy subjects between 20 and 29 years of age then underwent three rounds of ultrasonography at six different locations along the radial artery on each arm to estimate anatomical and hemodynamic properties. The six measurement locations consisted of the three PLs (*Cun*,* Guan*, and* Chi*) and three non-PLs that were more proximal than* Chi*.

### 2.1. Subjects

To focus on the differences between palpation locations, we selected a uniform subject group with the following inclusion criteria:

- those with a body mass index (BMI) in the normal range (18.5–23.0 kg/m³);
- those with no cardiovascular disease (hyperlipidemia, arteriosclerosis, coronary heart disease, stroke, etc.);
- those with no disease such as hypertension or diabetes nor taking any type of medication;
- those who had no surgery on the radial artery nor vascular deformity;
- those who did not smoke within one hour or drink alcohol within six hours prior to the start of the experiment.

Table 1 shows the basic physiological characteristics.

### 2.2. Study Design

**Figure 1:**
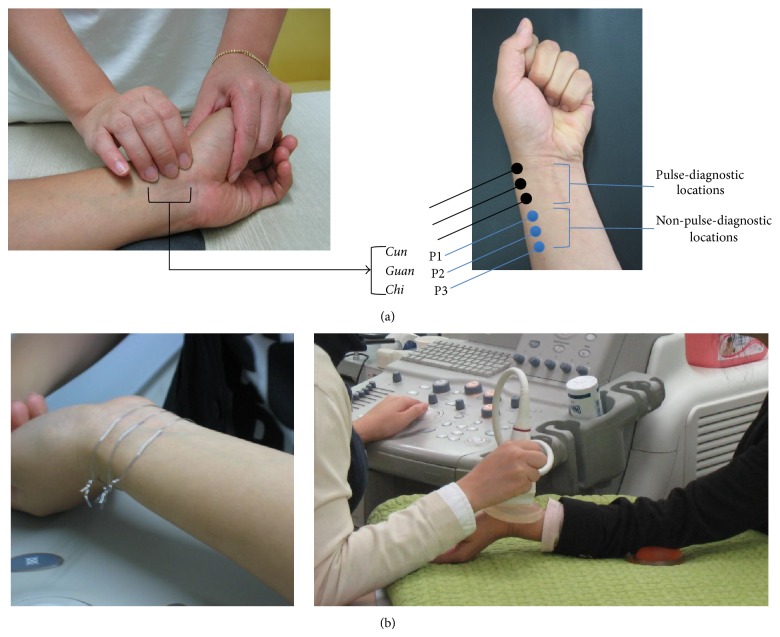
(a) Six measurement locations composed of the three pulse-diagnostic locations (*Cun*,* Guan,* and* Chi*) and non-pulse-diagnostic locations (P1, P2, and P3) on the radial artery and (b) marking of the measurement locations by thin wires (left) and a measurement scene (right).

After volunteers were deemed suitable, the arterial characteristics of each subject were determined using an ultrasound scanner. A TKMD then identified the accurate locations of* Cun*,* Guan*, and* Chi* and marked them. The three non-PLs of P1, P2, and P3 were marked equidistantly from* Chi* towards the elbow with a scale set by the distance between* Guan* and* Chi* as in Figure 1(a). We examined the arterial properties of 12 different locations for each subject (6 locations on each arm). To reduce the measurement bias, we randomized the sequence of the measurement locations for each subject and a single trained operator conducted all the measurements. To recognize these locations in the B-mode image on the ultrasound scanner's screen, we wrapped a tiny metal wire around each measurement location and placed a gel pad over each wire (Figure 1(b)). We then estimated the diameter and depth of the artery as well as the velocity of the blood flow at each measurement location using the ultrasound images. We observed that these values varied periodically due to the heartbeat; therefore, we froze the B-mode to the diastolic period for the analysis to provide equal conditions for individual subjects. To identify the diastolic period, we employed a photoplethysmograph (PPG) on the index finger of the subject during the ultrasonography and selected the lowest valley in the photoplethysmogram. The measurements were repeated three times at each location.

### 2.3. Devices and Measurement Technique

For the ultrasonography, a medical ultrasound scanner (Volusion 730 Pro, GE Medical, USA) was used and a 12L Probe was selected. The diameter and depth of the artery were measured in the B-mode and the blood flow velocity was measured in Color Doppler mode. The angle of incidence for the ultrasound was kept at 20° in Color Doppler mode and 60° in B-mode. A gel pad (Parker Laboratories, Inc., USA) was used for ease of measurement. The PPG signal was obtained using a Biopac PPG100C (from Biopac Systems, Inc., USA) to monitor real-time blood dynamics due to the periodic pumping of the heart.

### 2.4. Statistical Methods

We analyzed the data using SPSS 14.0 software (SPSS Inc., USA). We examined the mean differences in the diameter and depth of the artery and in the velocity of the blood flow using a 3-way repeated measures ANOVA test. Scheffe's post hoc test was employed when significant differences were found by ANOVA, and differences between pairs of left and right palpation locations, for example, between the left* Guan* and the right* Guan*, were examined using a paired* t*-test. The statistical significance was set at *P* < 0.05.

## 3. Results

We compared the mean values and standard deviations (SD) of the diameters and depths of the arteries as well as the maximum and average blood flow velocities at* Cun*,* Guan*,* Chi*, P1, P2, and P3 in the left and right arms. These data are summarized in Table 2 and Figure 3. The artery at* Guan* was located closest to the surface of the skin; and the artery at* Cun* was located between that of* Guan* and* Chi*, and it was monotonically deeper than that at* Guan* and superficial to that at P3. The arterial diameter was smallest at* Guan*, but it did not vary significantly between the measurement locations. The average and maximal blood flow velocities were substantially decreased at* Cun* compared to* Guan*, and these velocities gradually decreased from P3 to* Guan*.

ANOVA revealed significant differences in all of the anatomical and hemodynamic properties between the different measurement locations in both arms (*P* < 0.001 for the arterial diameter, depth, and average blood flow velocity (see Table 3)). We completed Scheffe's multiple comparisons test for the quantities that showed significant differences in ANOVA. As summarized in Table 3, each quantity was classified into one or more Scheffe's groups based on the measurement locations. All of the variables at* Guan* were distinctively grouped regardless of which side of the body was tested, implying that* Guan* represented a unique location.

We normalized the diameter and depth of the artery to the diameter of the arm at each measurement location to determine if the arterial depth and diameter depended on arm size. ANOVA revealed that the normalized artery depth differed significantly between* Cun*,* Guan*, and* Chi* (left: *P* = 0.001; right: *P* < 0.0001); however, no significant differences were observed among P1, P2, and P3. In contrast, the normalized artery diameter exhibited no significant differences between* Cun*,* Guan*, and* Chi*, but a significant difference was found among P1, P2, and P3 (left: *P* = 0.0002; right: *P* = 0.0032). These findings indicate that the arm diameter and artery depth at each location were not correlated.

Table 4 compares the arterial depth and diameter as well as the average and maximal blood flow velocities between the two arms using a paired* t*-test. All of the quantities were significantly different between the left and right sides; the arterial depth differed at* Cun*, P2, and P3, and the diameter differed at* Chi*, whereas the average and maximal blood flow velocities differed significantly at all measurement locations.

## 4. Discussion

The artery was closest to the skin surface at* Guan*, and it became monotonically deeper as it approached the elbow. Based on Scheffe's grouping, the artery at* Cun* was at a similar depth (left arm) or deeper (right arm) than the artery at* Chi*, and this result was similar to that from our preliminary ultrasonographic study, which showed that the artery was deeper in the order of* Guan*,* Chi*, and* Cun* for both male and female subjects [9]. Normalizing the artery depth to arm circumference at the measurement location revealed a significant mean difference between* Cun*,* Guan*, and* Chi*; however, the original differences between the non-PLs of P1, P2, and P3 were found to be nonsignificant after normalization. This result implies that the artery depth along P1, P2, and P3 is proportional to arm size, but that modulation of the arterial depth around the three PLs results from more complex origins, such as the bony structure at the styloid process (see Figure 4).

A difference in blood flow causes changes in blood pressure that are proportional to the pressure energy [10]. If other variables, such as depth and the compliance of the blood vessel, are identical between two measurement locations, then the measured pulse power will be proportional to the pressure energy. Assuming that blood viscosity is constant and that the blood vessel is rigid, we can apply Bernoulli's equation to obtain the blood pressure difference between two measurement locations, for example, between locations *A* and *B*:

(1) $$\begin{array}{c} P_{A}-P_{B}=\rho \times \frac{\left(v_{B}^{2}-v_{A}^{2}\right)}{2}, \end{array}$$

where *ρ* is the blood density and *v*_*A*,*B*_  is the blood flow velocity at location *A* or *B*. Using the results outlined in Table 2 and *ρ* = 1.06 × 10^−3^ kg/cm^3^ for blood viscosity, the pressure increase at* Cun* due to the velocity decrease relative to* Guan* is maximally given by *P*_*Chon*_ ≈ *P*_*Gwan*_ + (4.9 ± 0.3) mmHg. When using this equation, the velocity gradient between* Guan* and* Chi* is an order of magnitude smaller than that between* Guan* and* Cun*, which yields *P*_*Gwan*_ ≈ *P*_*Cheok*_ + 0.4 mmHg. In addition, the pressure differences between non-PLs were not negligible (in sub-mmHg units). These features were common to both the left and right arms.

For a healthy individual, the systolic blood pressure is approximately 100 mmHg. Therefore, difference in the maximal pressure gradient between* Cun* and* Guan* (Δ*P* ≈ 5 mmHg) is physiologically meaningful. Similar deviations were not observed for other locations which addresses two major topics. First, a wrist-worn blood pressure monitor should avoid a* Cun* location to minimize uncontrolled physiological changes and to improve repeatability. Second, the average pulse power at* Cun* may appear slightly higher when compared with other palpation locations. To estimate the effect of the blood pressure gradient on the pulse power or tension of the artery, the energy dissipation rate through the skin and the response of the artery to the contact pressure must be determined; however, this is beyond the scope of this work.

The diameter and depth of the artery in addition to the blood flow velocity were significantly different between the left and right palpation locations, and the blood flow velocity showed the largest significant differences. For instance, the maximal flow in the left radial artery was approximately 5 cm/sec faster than in the right artery, with small variations depending on the measurement location. This variation may have resulted from differences in the anatomical location of the heart and of the muscle development of the dominant hand [11, 12]. According to King et al., the pulse strength measured at the right brachial artery was relatively high when compared with the left arm for both genders [13]. A future study designed to synchronously measure blood flow and pulse strength needs to be completed to understand the characteristics of and the blood flow in the left and right radial arteries.

We did not address gender differences in this study. Indeed, male subjects exhibited a higher blood flow velocity, larger artery diameter, and shallower artery depth when compared with female subjects. However, the overall tendencies of the blood flow velocity, artery diameter, and depth across the three PLs and three non-PLs were identical between male and female subjects. We observed the same tendencies after combining both gender groups. Therefore, we only presented the combined data from both genders.

**Figure 2:**
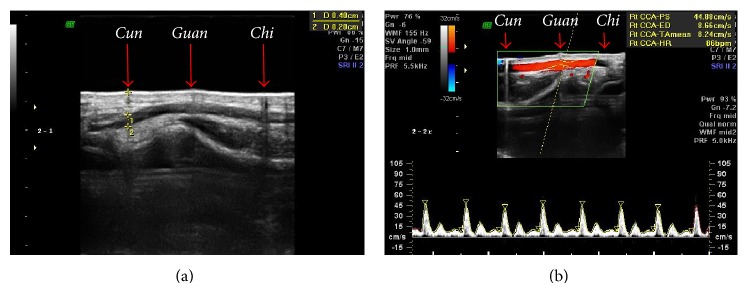
(a) The measurement of the arterial depth and diameter in B-mode and (b) the measurement of the blood flow velocity in the Color Doppler mode.

**Figure 3:**
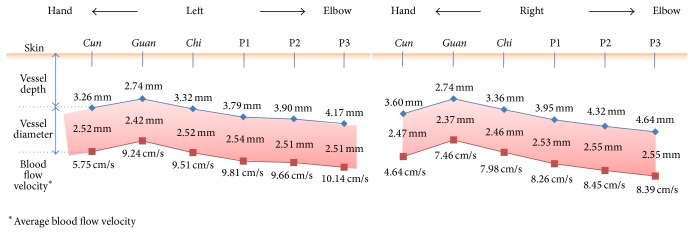
Diagram of the anatomical and hemodynamic characteristics at both arms.

**Figure 4:**
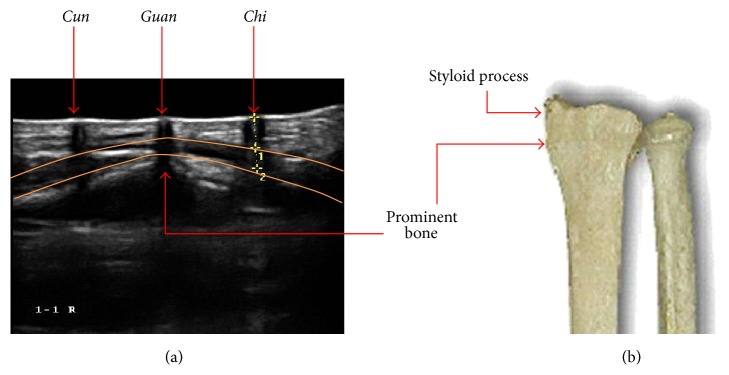
(a) Ultrasonographic image which shows the changes in the vessel features around the pulse measuring locations and (b) it shows the detailed shape of the prominent bone beneath* Guan*.

We found that arm posture affected the artery depths at* Cun* and* Guan*; however no such dependence was found at* Chi* or at the three non-PLs. Similarly, Chung et al. reported that the first pulse appearances at* Cun* or* Guan* highly depended on the pulse-taking posture [14, 15], in which they kept the artery levels at the* Cun-Guan-Chi* at the same altitude to acquire repeatability in the depth of the first pulse appearance. On the other hand, in this study, we maintained a constant altitude for the skin surface levels at* Cun* and* Guan* (see Figure 2), which was a natural pulse-taking arm posture used by many pulse practitioners in clinics.

We believe that our arm-posture technique and the arm posture used by Chung et al. are suitable for reliable pulse reading. However, we emphasize that maintaining the artery level at the same altitude cannot circumvent the blood pressure gradient between* Cun* and* Guan,* as claimed by Chung et al. [15]. The blood pressure gradient near* Cun* does not occur due to the differences in gravitational force between the* Cun* and* Guan* locations. Instead this gradient occurs naturally by the abrupt increase of the blood flow resistance at the entrance of the palm (see (1)) [16]. A simple calculation shows that the pressure gradient between* Cun* and* Guan* due to the gravitational force that occurs after a change in arm posture is negligible (on the order of 10^−4 ^mmHg or less).

The pulse is a pressure wave that is related to but distinct from blood flow [10]. Therefore, the results discussed here can give only a partial explanation of pulse diagnostics. Investigations on different ages and diverse health conditions may result in divergent outcomes. More advanced study design that compares pulse diagnoses and ultrasonography will reveal more significant results that can verify some of fundamentals on pulse diagnostics; for instance, an explicit relation between pulse properties and hemodynamic/anatomical properties at* Cun, Guan*, and* Chi* can be obtained. Relation between measuring arm posture and pulse features or arterial features is another research topic that will help standardize the pulse reading posture.

## 5. Conclusions

We studied the anatomical and hemodynamic properties of the radial artery, including the artery diameter and depth as well as the average and maximum velocities of the blood flow at the three PLs and three nearby non-PLs. We selected 44 subjects aged 20–29 years and examined their diastolic pulse period using an ultrasound scanner. We found that the radial artery was most superficial at* Guan* (prominent bone) and that it became monotonically deepened as it moved towards the elbow. The maximal and average blood flow velocities changed substantially in the PL regions, while they only changed slightly at the non-PL regions. All of the investigated measurements contributed to the characteristics of the pulse. Therefore, the substantial modulation of the anatomical and hemodynamic properties of the radial artery across the PLs and the disappearance of these modulations at the nearby non-PLs partially explain the diagnostic use of* Cun*,* Guan*, and* Chi*.

## Figures and Tables

**Table 1:** Subjects' information.

Subject information	Mean ± SD
Number (*n*)	23 males and 21 females
Age (yr)	23.17 ± 2.16
Height (cm)	167.26 ± 8.04
Weight (kg)	59.04 ± 7.84
BMI (kg/m^2^)	21.01 ± 1.27
Systolic/diastolic blood pressure (mmHg)	115.80/70.19 ± 15.15/8.29
Total cholesterol (mg/dL)	165.40 ± 30.91

**Table 2:** Artery depth and diameter and blood flow velocities.

Variable	Measurement location [mean (SD)]					
	*Cun *	*Guan *	*Chi *	P1	P2	P3
Left						
Artery depth (mm)	3.26 (0.71)	2.74 (0.66)	3.32 (0.85)	3.79 (1.09)	3.90 (1.11)	4.17 (1.26)
Artery diameter (mm)	2.52 (0.36)	2.42 (0.35)	2.52 (0.30)	2.54 (0.32)	2.51 (0.31)	2.51 (0.31)
Maximum blood flow velocity (cm/sec)	41.68 (12.39)	55.34 (14.70)	56.26 (11.82)	54.98 (12.23)	56.36 (12.21)	57.66 (13.57)
Average blood flow velocity (cm/sec)	5.75 (3.25)	9.24 (4.86)	9.51 (4.44)	9.81 (5.24)	9.66 (4.51)	10.14 (5.04)
Right						
Artery depth (mm)	3.60 (0.82)	2.74 (0.72)	3.36 (1.14)	3.95 (1.36)	4.32 (1.42)	4.64 (1.41)
Artery diameter (mm)	2.47 (0.39)	2.37 (0.32)	2.46 (0.33)	2.53 (0.41)	2.55 (0.34)	2.55 (0.33)
Maximum blood flow velocity (cm/sec)	35.50 (4.64)	49.28 (11.95)	50.91 (11.55)	51.43 (12.53)	52.85 (13.15)	53.94 (12.13)
Average blood flow velocity (cm/sec)	4.64 (2.99)	7.46 (4.24)	7.98 (4.41)	8.26 (4.44)	8.45 (5.12)	8.39 (4.43)

**Table 3:** Results of ANOVA and multiple comparison analysis.

		ANOVA	Scheffe's grouping					
		*P* value	*Cun *	*Guan *	*Chi *	P1	P2	P3
Left	Artery diameter	<0.0001	A	B	A	A	A	A
	Artery depth	<0.0001	A	B	A	C	D	E
	Average blood flow velocity	<0.0001	A	B	B C	B C	B C	C

Right	Artery diameter	<0.0001	A	C	A	A B	B	B
	Artery depth	<0.0001	A	B	C	D	E	F
	Average blood flow velocity	<0.0001	A	B	C	C	C	C

Each letter from A to F indicates distinct group by Scheffe's test.

**Table 4:** Paired *t*-test between the left and right arms.

	Artery depth	Artery diameter	Average blood flow velocity	Maximum blood flow velocity
L_*Cun*-R_*Cun *	0.004^**^	0.329	0.008^**^	0.000^**^
L_*Guan*-R_*Guan *	0.987	0.224	0.001^**^	0.000^**^
L_*Chi*-R_*Chi *	0.740	0.049^*^	0.003^**^	0.000^**^
L_P1-R_P1	0.169	0.852	0.005^**^	0.000^**^
L_P2-R_P2	0.000^**^	0.253	0.011^*^	0.000^**^
L_P3-R_P3	0.000^**^	0.299	0.001^*^	0.017^*^

L: left, R: right, ^*^ *P* < .05, and ^**^ *P* < .01.
